# A National Misfortune

**Published:** 1897-01

**Authors:** 


### EDITORIAL.

### A NATIONAL MISFORTUNE.

THE resignation of Prof. F. H. Osgood, Chairman of the Board of Cattle Commissioners of Massachusetts, is a misfortune of national importance. Not that the Bay State does not possess other members of the profession who can carry out the provisions of the law, but for the very grave reason that Prof. Osgood felt bound to withdraw from this important work because he was not willing to spend public money appropriated for scientific work in the interests of public health when the plans and measures for its wise use have been surrounded with such unwise restrictions as practically to subvert the great purpose of this movement. It is not often that our public servants have such high ideas of true responsibility in the spending of public money raised by general taxation, and these restrictions are a step backward for this great centre of educational progress, and no one can foretell the end. It is a serious menace to the progress of sanitary police work in this country, because our whole nation at certain epochs in the history of animal plagues has waited for the results obtained in Massachusetts, as that State set the pace in broad and advanced methods for dealing with the trouble. No one will question the strong grounds of action for this self-retirement by Dr. Osgood, but all with one accord regret that such conditions exist to cause so great a loss at this time. Entering upon this work at great personal sacrifice, Dr. Osgood, as Chairman of this Board, grasped fully the momentous character of this question of bovine tuberculosis and presented the most comprehensive and advanced plans for the control, eradication, and future protection from its losses ever offered in any nation or State. While we have always expected much from the people of that great commonwealth, and have reaped rich harvests from its many contributions to the public welfare, it should be said that never in the history of mankind did a State or people enter upon a sphere of work of as much vital importance with as well-laid plans as the movement set on foot to deal broadly and effectually with this scourge of the world, and which promised more true light and benefit to public health than any similar movement ever inaugurated. We profoundly regret this unexpected loss, which will halt in greater or less measure every movement for wiser and better sanitary police measures in every centre of our land, and will do much to deter action in many States where similar plans would have early matured for the conservation of this great problem.

In conclusion, may we ask of the higher officers and people of this great State, Can you afford to halt a movement of so much importance to the health and happiness of the people of the whole earth?

*What will your answer be?*

